# The Role of Primary Repair of the Recurrent Laryngeal Nerve during Thyroid/Parathyroid Surgery in Vocal Outcomes—A Systematic Review

**DOI:** 10.3390/jcm12031212

**Published:** 2023-02-03

**Authors:** Elisavet Papadopoulou, Konstantinos Sapalidis, Stefanos Triaridis, Athanasia Printza

**Affiliations:** 1 1st Department of Otorhinolaryngology, School of Medicine, Faculty of Health Sciences, Aristotle University of Thessaloniki, 54124 Thessaloniki, Greece; 2 3rd Department of Surgery, School of Medicine, Faculty of Health Sciences, Aristotle University of Thessaloniki, 54124 Thessaloniki, Greece

**Keywords:** recurrent laryngeal nerve injury, paresis, primary repair, vocal outcome, voice, thyroid surgery, parathyroid surgery, outcome measures, neurorrhaphy

## Abstract

Recurrent laryngeal nerve (RLN) injury is a well and long-known complication of thyroid and parathyroid surgery that significantly affects the quality of life of patients. Despite the advances in surgical techniques and technology, it still occurs in clinical practice either as temporary paresis or as permanent paralysis of the corresponding vocal cord. The purpose of the current systematic review is to examine the value of intraoperative repair of the RLN in voice restoration. A systematic review of the existing literature was conducted using PubMed, Scopus, Cochrane Library, and Google Scholar databases according to the PRISMA guidelines. The systematic review resulted in 18 studies, which met the inclusion criteria. An improvement in phonatory function and voice quality was observed in all these studies after immediate RLN reconstruction (not always statistically significant). This improvement appears to be comparable to or even higher than that achieved with other methods of repair, and in some cases, the improvement approaches levels found in normal subjects. Intraoperative RLN reconstruction is not widely used in clinical practice, but the evidence so far makes it a viable and safe alternative to traditional techniques with better long-term results, as it prevents the occurrence of atrophy of the vocal cord and should be considered in the operating room if possible.

## 1. Introduction

Injury of the recurrent laryngeal nerve (RLN) during thyroid and parathyroid surgery is a well-known complication and a common cause of malpractice accusations as it highly affects the quality of life (QoL) of patients [1]. Despite the optimism being brought by the advances in surgical techniques, a better understanding of anatomy and physiology, and the use of neuromonitoring intraoperatively, the rates of RLN palsy have not decreased significantly [2,3,4,5,6,7,8,9]. More specifically, the rates of ipsilateral vocal cord permanent paralysis have been reported to range from 0.1 to 5% (most studies report rates 0.3–3%) and those of transient palsy have been reported to reach up to 8% depending on the institution [1,10].

RLN injury affects all three main functions of the larynx: phonation, protection of the airway during swallowing, and, of course, breathing [11,12]. Isolated injury of the superior laryngeal nerve can also occur, affecting the voice and swallowing. The most common clinical finding after the injury of the RLN in thyroidectomy is dysphonia, which may or may not coexist with some degree of dysphagia and even aspiration due to the paralysis of the corresponding vocal cord (VC) [13]. The voice in these cases is described as weak with easy fatigue and breathiness. Very often, it is also accompanied by a subjective feeling of shortness of breath during phonation. This situation negatively affects the QoL of the patients and is one of the most common causes of legal disputes following thyroid surgery [13]. If the damage involves both RLNs, then the predominant symptom is shortness of breath and even respiratory failure with stridor already seen in the operating room and resuscitation [14].

RLN resection can occur either inadvertently or deliberately (for oncological purposes, such as in advanced loco-regional cancer invasion). The management options for RLN injury/transection include both conservative and surgical methods. The treatment is determined by many factors, such as the time that the injury is being noticed (intra-operatively or post-operatively), severity of symptoms, vocal fold positions, overall disease prognosis, and, of course, patient history, age, vocal needs, and wishes [13]. The treatment options are voice therapy, medical treatment (steroids), injection laryngoplasty, medialization procedures (thyroplasty type 1 with or without arytenoid adduction), reinnervation procedures (primary or secondary), nerve–muscle pedicle transplantation, and electrical stimulation of the recurrent laryngeal nerve [15,16,17]. Although several medialization procedures improve vocal quality by decreasing the glottal gap during phonation, they cannot prevent the forthcoming vocal fold atrophy that compromises the long-term results. Neurorrhaphy on the other hand aims to restore the integrity of the RLN and preserve normal muscle tone. After neurorrhaphy, nerve regeneration does happen but in a random, misdirected way resulting in simultaneous contraction of the abductor and adductor muscles [18,19]. Normal motion is not restored, but no atrophy occurs, because the normal tension and volume of the vocal cords are restored, resulting in voice improvement. Recently, the American Association of Endocrine Surgeons in its 2020 guidelines strongly recommends “that if RLN transection is detected intraoperatively an attempt should be made to repair it” [20].

The purpose of this paper is to review systematically and critically the current evidence of the role of immediate neurorrhaphy of the RLN as a repair method of RLN injury, in vocal outcomes and to provide evidence to all thyroid surgeons of the optimal approach during the operation. The present study is a systematic review of the literature surveying the value of early intra-operative reinnervation in the restoration of RLN damage during thyroid and parathyroid surgery, especially in terms of voice quality, aiming to fill the knowledge gap on the role of early reinnervation of the RLN in voice improvement. The primary endpoint is the improvement of the voice. Additionally, the potential superiority of one reinnervation method over the others will be investigated, as well as whether the presence of pre-operative vocal cord paralysis (VCP) affects the result.

## 2. Materials and Methods

### 2.1. Search Strategy

**Figure 1:**
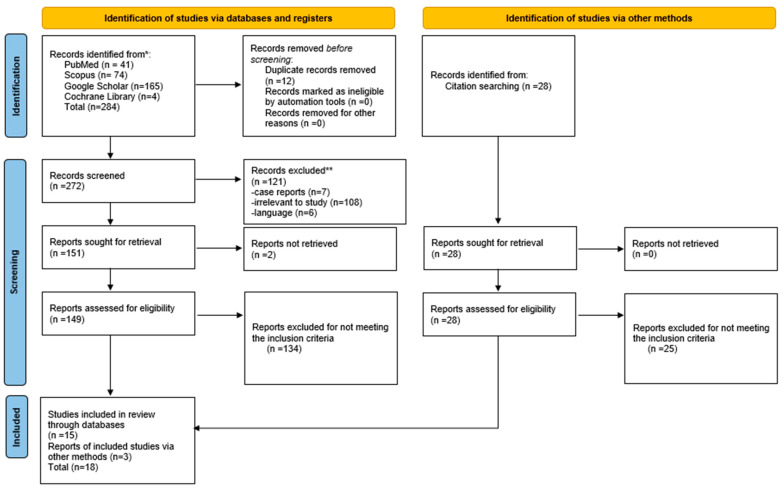
PRISMA flow diagram.

The systematic review was performed according to PRISMA guidelines [21] and the latest update [22] (see Supplementary Materials). A thorough search of the existing literature from inception to June 2022 was conducted on four different databases: PubMed, Scopus, Cochrane Library, and Google Scholar by two independent researchers (EP and AP). Researchers discussed with experts with an interest in the subject under study, to obtain any unpublished or ongoing studies and to resolve any possible disagreement. For updating the searches, email alerts were used as method for rerunning the searches until the end of June of 2022. The keywords used for the search were a combination of the following terms: immediate, primary repair, reinnervation, neurorrhaphy, recurrent laryngeal nerve, thyroid surgery, parathyroid surgery, and thyroidectomy. The term vocal outcome was not added in the search as it minimized the results in every database. Nevertheless, exactly this vocal outcome, was searched in every study sought for retrieval so as to assess the eligibility of each study for this systematic review. The flowchart of this search is presented in Figure 1, documenting the total number of records identified from each database and other methods (citation searching).The exact search terms in each database are presented in Table 1.

### 2.2. Inclusion and Exclusion Criteria

Studies on adults (≥18 years) reporting primary, intraoperative reinnervation of RLN in patients who underwent thyroid and/or parathyroid surgery for benign or malignant disease, either as first surgery or as a re-operation were included. Studies meeting the above criteria were included regardless of the presence of pre-operative VCP, the method of voice function evaluation used, or the type of neurorrhaphy chosen. Any non-English language studies were excluded. Animal trials, oral presentations, case reports, and reviews were also excluded. Because the present study is a review of already published studies, no hospital scientific board and bioethical committee approvals were required, and no consent of any kind was required. A summary of the inclusion and exclusion criteria is presented in Table 2, using the PICO strategy.

### 2.3. Quality Assessment of the Studies Included

None of the studies included were randomized. For a better and more thorough assessment of the non-randomized studies included in this review, two different risk of bias assessment tools were used for every study, which contained different domains of assessment. The assessment was conducted by two independent reviewers. The rationale for using two different tools for quality assessment was to provide as many details of the studies selected in this review. For instance, with the Newcastle–Ottawa scale, emphasis was given on the adequacy of the follow-up period and whether it was long enough for the outcomes to be safely assessed, while with ROBINS-1, the most frequently used tool for quality assessment, bias concerning missing data and or any deviation of the intended intervention were assessed. First, Cochrane’s collaboration tool, “Risk Of Bias In Non-randomized Studies of Interventions”, ROBINS-I [23] was used, a tool that covers seven distinct domains through which bias might be introduced (The scoring is “Low risk”, “Moderate risk”, “Serious risk”, and “Critical risk” of bias for each domain). The results were presented using robvis, a web app designed for visualizing risk of bias assessments performed as part of a systematic review. The other tool for quality assessment of non-randomized studies that we used was the Newcastle–Ottawa scale [24]. This assessment scale contains eight items in three domains, specifically bias concerning the selection process, the comparability of the cohorts, and the assessment of outcome, with a total maximum score of nine stars. A study with scores from 7 to 9 has high quality, 4–6, high risk, and 0–3 present a very high risk of bias. Two reviewers assessed each study using both the above tools, working independently. The results of this assessment were discussed thoroughly by all reviewers, and if any disagreement occurred, all reviewers reassessed the studies.

### 2.4. Statistical Analysis

The ultimate purpose of this work was to perform a meta-analysis after synthesizing and processing the data, both on a quantity and a quality level. However, the small number of studies (only 18 met the inclusion criteria), the lack of some data in many studies (for instance, the exact time of the post-op examination reported on text, despite attempts to collect the missing information via contacting the authors), and the differences in the study protocol (completely different ways of assessing the post-op vocal outcomes in most of the studies) combined with the large heterogeneity in the way of presenting the data (some only displayed mean values compared to every single measurement of each patient, other authors separated the presentation of results by gender), as well as the different post-operative follow-up intervals, made it impossible to perform a meta-analysis. Moreover, even at the attempt to compare all studies that used MPT (14/18) to assess the vocal quality, only 6 studies out of 14 present mean values at 1 year post-op, and yet, the male to female ratio is quite different.

## 3. Results

A total of 18 articles met the inclusion criteria and were selected in this review (Figure 1). The studies and their characteristics are presented in Table 3 including the demographic characteristics of the populations, the sample size of each study, and the methods used to evaluate the vocal results. Regarding the methodology of the studies, 16.7% (*n* = 3) are prospective comparative studies, 11.1% (*n* = 2) are prospective studies, 45.5% (*n* = 8) are retrospective comparative studies, and 27.7% (*n* = 5) are retrospective studies. No randomized clinical trials (RCTs) were found. The duration of the post-operative follow-up shows great heterogeneity, from 6 months to 15 years after surgery. Only 3 of the 18 studies compare the vocal outcome according to the presence or absence of pre-operative UVFP. In 8 out of the 18 studies, a comparison was made between different techniques of neurorrhaphy of the RLN. As for the method of evaluating the vocal function, great heterogeneity was also found. Some studies report only subjective methods (*n* = 4). Most articles originate from Asian countries (*n* = 15, 83%) with Japan being the predominant country of origin (*n* = 7). South Korea follows with three studies; two studies are from China; one is from Taiwan, and one is from Malaysia. Only three originated from Europe and the USA.

To summarize the studies included in this systematic review, reinnervation can be performed with four different methods: direct anastomosis (DA), when the deficit of the transected RLN is less than 5 mm; ansa to recurrent nerve anastomosis (ARA), using ipsilateral or contralateral ansa cervicalis (the anastomosis can be made either outside of the thyroid cartilage or inside of the thyroid cartilage); vagus to recurrent nerve anastomosis (VRA), whenever both RLN and vagus nerve must be sacrificed, and with the use of free nerve graft (FNG). In the case of FNG, two anastomoses are required, and the most common nerve donors are the greater auricular nerve, ansa cervicalis, transcervical nerves, and supraclavicular nerves.

The methods of voice evaluation used in the studies included showed great heterogeneity, with some studies reporting only subjective methods (n = 4). Videostroboscopy, Laryngoscopy, Maximum Phonation Time (MPT), Phonation Efficacy Index (PEI: a sex-independent indicator of vocal function defined by MPT divided by the vital capacity), Maximum Flow Rate (MFR), Acoustic Analysis (Harmonics to Noise Ratio—HNR, jitter, and shimmer), Laryngeal Electro-Myography (LEMG), and questionnaires, such as the Voice Handicap Index (VHI), and the perceptual evaluation of the voice by the examiner according to the Grade–Roughness–Breathiness–Authenticity– Strain Scale (GRBAS scale) were used for post-op voice evaluation. All but one study used an endoscopic evaluation of the larynx (laryngoscopy or/and videostroboscopy). The second most frequently used assessment method was MPT, which was used in 14 studies (77.8%). Only two studies used laryngeal electromyography. For patient-reported outcomes, the tool of choice was VHI, either in its extended 30-question form or the short version of 10 questions. Voice perceptual evaluation was also used with the GRBAS scale and the Hirano scale, a short version of the GRBAS scale. Aspiration assessment was conducted only in two studies, specifically, both studies used a 4-point scale for aspiration rating (0: none–3: severe), which was filled out by physicians according to the patient’s report.

Table 4 presents the results and conclusions of the studies included in the systematic review. With primary reinnervation of the larynx, it seems possible to achieve satisfactory phonation while maintaining normal tone and volume of the vocal cord and preventing the appearance of atrophy because the normal resting tone is maintained in the reinnervated muscles. It is worth noting that serious complications did not present in any of the studies in the review, and the voice results were judged to be satisfactory and comparable to a general population in all of them, while they were clearly superior to other methods of RLN injury repair.

### Risk of Bias

**Figure 2:**
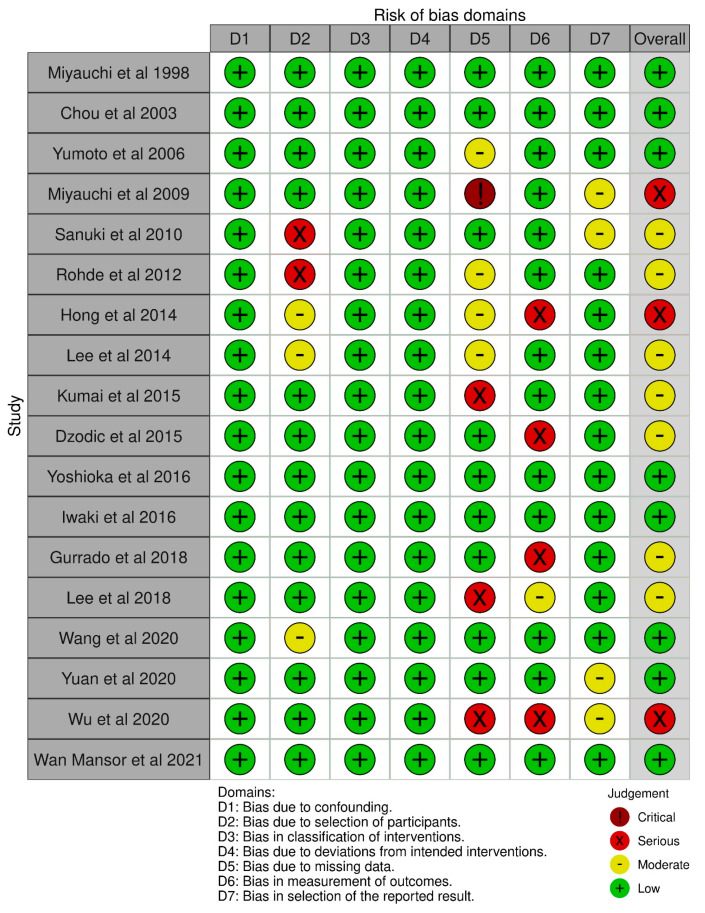
Risk of bias of the studies included individually [18,25,26,27,28,29,30,31,32,33,34,35,36,37,38,39,40,41].

**Figure 3:**
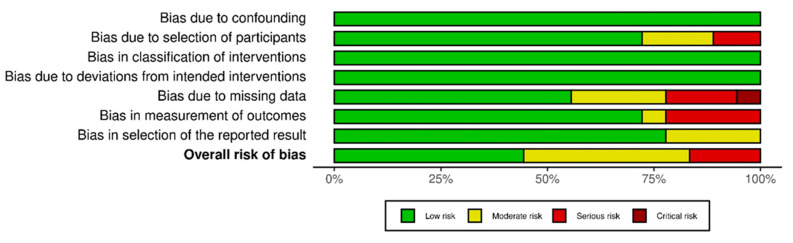
Overall presentation of risk of bias of the studies included.

Regarding the risk of bias assessment of the comparative studies, the use of the Newcastle–Ottawa scale showed a score from six to eight in eleven studies, while five scored eight stars. As for non-comparative studies, seven studies scored from five to seven. The exact scoring for every category is shown in Table 5. The weakest assessment point was found in the assessment of the outcome and the adequacy of the follow-up period. The use of Cochrane’s collaboration tool, ROBINS-I showed an overall low and moderate risk of bias and is presented in Figure 2 and Figure 3 (individually and overall). The weakest assessment points concerned biases in the measurement of outcomes, in selection of the reported results, and some missing data.

## 4. Discussion

The idea of immediate neurorrhaphy of the transected RLN is very simple, and in theory, it appears to be the most logical and appealing method of repair; consequently, the first reports go back more than a century. The first successful attempt of direct anastomosis was reported by Horsley in 1910 with complete restoration of movement a year after the surgery, followed by other reinnervation procedures using various techniques, such as ansa cervicalis to RLN anastomosis (Frasier, 1926) [42], vagus to RLN anastomosis (College Balance, 1927), and free nerve graft (Berendes Miehlke, 1968) [11]. Despite these first favorable results, all the later studies failed to report vocal movement restoration. Consequently, reinnervation was abandoned as a repair method. The last two decades, a renewed interest has been encountered in the literature with many new publications. This change of scenery was prompted by the studies of Ezaki et al. [43], Crumley et al. [44], and Miyauchi et al. [25], which reported favorable vocal outcomes even though the vocal cord remained fixed in the median or paramedian positions. The RLN is a motor nerve that consists of both adductor and abductor nerve fibers. After neurorrhaphy, nerve regeneration does happen but in a random, misdirected way resulting in simultaneous contraction of the abductor and adductor muscles [19].

To summarize the findings of the studies included in this review, the restoration of normal movement was not achieved, but no atrophy was encountered either. A minimal or no glottal gap during phonation was observed with the restoration or improvement of the mucosal wave characteristics post-op. Indexes, such as MPT and PEI, improved with the improvement being noticed 2–5 months post-operatively. Finally, an improvement was reported in the acoustic analysis and the GRBAS and VHI scales.

This systematic review elucidates four key points regarding the role of the primary neurorrhaphy of the RLN in the repair of its injury during thyroid and parathyroid surgery. Specifically: (1) Intraoperative reinnervation of the RLN appears to improve phonation post-operatively. (2) This improvement is comparable to or superior to that achieved by other more widely used methods of vocal cord palsy repair and in some cases the levels found in a healthy control population. (3) There does not seem to be superiority of one method of neurorrhaphy over another in terms of post-operative vocal improvement. (4) The existence of a pre-operative paralysis of the vocal cord does not seem to affect the post-operative results in terms of the improvement of phonation after neurorrhaphy.

In summary, an improvement in post-op phonation in almost every parameter used for its evaluation is observed in all studies included. This is not always accompanied by statistical significance (*p* < 0.05). It must be noted that every study used a different post-op voice evaluation protocol for a different follow-up period. This highlights the need to establish core outcome sets (COSs) as an agreed-upon standardized set of outcomes that should be measured and reported, at a minimum, in all clinical trials in specific areas of health [45].

All the studies included in the research indicate favorable results regarding phonation after immediate repair. There does not seem to be any superiority of one method over another, except for the time needed to achieve the improvement. The existence of pre-operative ipsilateral VC paresis should not be a criterion for exclusion from the intervention, as favorable results were presented in both population groups. The surgical time increases, but there is no longer a need for intervention in the post-operative period with consequent lower costs and a better quality of life for the patient who does not have to undergo the discomfort and stress of a second surgery, while also avoiding the re-administration of some form of anesthesia. The surgical technique is simple with a small learning curve and simply requires the identification and preparation of the involved structures and then neuro-suturing with or without the use of optical magnification (microscope and loupes). Even in the case of an unsuccessful reinnervation, the remaining restorative techniques can still be used to improve the patient’s quality of life.

## 5. Conclusions

Intraoperative reinnervation of the RLN is not widely used, but so far, the data suggest that it is a viable and reliable alternative to traditional techniques with better long-term results. Recent reviews published by Masuoka et al. [46] and Simo et al. [47] reach the same conclusion. Every thyroid and parathyroid surgeon should be aware of this procedure and not hesitate to use it in the operation room at the time of RLN injury, as recommended by recent guidelines. In conclusion, it is noted that the combined use of early neurorrhaphy of the RLN with IL could be an ideal approach that bridges the 2–5-month time that must elapse for the favorable results of neurorrhaphy to appear while achieving the best long-term results that neurosurgery provides against IL. Large multi-center prospective studies, that will include larger series of patients and a control group using subjective and objective assessment methods with a longer post-operative follow-up, will help confirm the true value in a more reliable way and expand its use in the future.

## Figures and Tables

**Table 1:** Search terms used in search strategy.

Search Terms	
PubMed	All fields: immediate OR primary AND repair OR reinnervation OR neurorrhaphy AND recurrent laryngeal nerve AND thyroid surgery
Scopus	All fields: ALL (repair OR reinnervation AND recurrent AND laryngeal AND nerve AND immediate OR primary AND neurorrhaphy)
Cochrane Library	Title abstract keywords: recurrent laryngeal nerve AND primary repair OR neurorrhaphy AND vocal function
Google Scholar	All fields: (repair OR reinnervation or neurorrhaphy “recurrent laryngeal nerve” AND immediate OR primary)

**Table 2:** PICO strategy for the literature search.

Patients	Adults who underwent intra-operative reinnervation of RLN during thyroid or/and parathyroid surgery.
Intervention	Primary neurorrhaphy of RLN
Comparison	Other methods of RLN injury repair, or general population (without any known vocal dysfunction), or no comparison.
Outcome	Vocal function outcome (evaluated by any method)

**Table 3:** General characteristics of the included studies.

Authors, Year, Country	Study Period	Study Design	Sample	Method of Repair	Mean Age	Women	Methods of Assessment	Post-Op Follow-Up
Miyauchi et al., 1998, Japan [25]	1983–1995	RC	(a) 34, (b) 26, (c) 34 ^1^	ARA:19, FNG:8, DA:5, VRA:2	(a) 52	(a) 28, (b) 17, (c) 28	Laryngoscopy, MPT	>1 y, mean: 4.2 y
Chou et al., 2003,Taiwan [26]	1998–2001	RC	(a) 8, (b) 4 ^2^	DA:8	(a) 42.4 (b) 53	(a) 6, (b) 3	Videostroboscopy, Laryngoscopy, GRBAS, MPT	>6 m, mean: 10.5 m
Yumoto et al., 2006, Japan [27]	1995–2002	PC	(a) 9, (b) 9, (c) 4 ^3^	FNG:8, DA:1	(a) 57.8 (b) 54.4 (c) 20.5	(a) 7, (b) 6, (c) 2	Videostroboscopy, MPT, MFR, HNR, jitter, shimmer	>9 m, mean: 17 m
Miyauchi et al., 2009, Japan [18]	1984–2007	RC	(a) 88, (b) 34, (c) 27 ^4^	ARA:65, FNG:14, DA:7, VRA:2	(a) 56	(a) 72, (b) 26, (c) 18	Laryngoscopy, MPT, PEI	1 y
Sanuki et al., 2010, Japan [28]	2000–2008	RC	(a) 6, (b) 6 ^5^	FNG:9, ARA:2, DA:1	(a) 60.5 (b) 63.1	(a) 5, (b) 3	Videostroboscopy, MPT, MFR, GRBAS	>7 m, mean: 34.6 m
Rohde et al., 2012, USA [29]	2002–2012	P	9	DA:4, FNG:2, VRA:2	56.2	6	Videostroboscopy	>9 m
Hong et al., 2014, South Korea [30]	2004–2011	RC	(a) 10, (b) 4 ^6^	(a) DA:10. All patients had IL 2–6 m post-op	(a) 48.1 (b) 54.3	(a) 8, (b) 4	Laryngoscopy, aspiration assessment, GRBAS, VHI, MPT	>1 y, (a) m mean (a/b): 19.6/19.3 m
Lee et al., 2014, South Korea [31]	2008–2012	P	19	DA:12, ARA: 7	48.4		Videostroboscopy Laryngoscopy, MPT, HNR, jitter, shimmer, VHI-30	2 y (0.6 m, 12 m, 24 m)
Kumai et al., 2015, Japan [32]	2000–2011	R	17	FNG:9, ARA:8	60.5	12	Videostroboscopy, MPT, MFR, jitter, shimmer	1 y
Dzodic et al., 2015, Serbia [33]	2000–2015	R	16	ARA:11, DA:5			Laryngoscopy, vocal assessment qualitative scale (0–5)	1 y
Yoshioka et al., 2016, Japan [34]	1998–2014	RC	(a) 449, (b) 1257, (c) 40 ^7^	ARA:345, DA:59, FNG:35, VRA:10	(a) 59.2	(a) 354 (b) 1045 (c) 29	Laryngoscopy, MPT, MFR	1 y
Iwaki et al., 2016, Japan [35]	2009–2011	PC	(a) 13, (b) 8, (c) 14 ^8^	(a+b) ARA:17, FNG:3, DA:1	(a+b) 60.6 (c) 65.9	(a) 10, (b) 7, (c) 3	Laryngoscopy. MPT, PEI, jitter, shimmer, HNR	(a+b) 1 y (c) 3 m
Gurrado et al., 2018, Italy [36]	2000–2015	RC	(a) 5, (b) 7 ^9^	DA:5	(a) 57.4 (b) 56.8	(a) 3, (b) 5	Videostroboscopy, GRBAS, aspiration assessment, subjective vocal assessment scale	>9 m
Lee et al., 2018 South Korea [37]	2005–2016	PC	(a) 19, (b) 43 ^10^	ARA:12, DA:7	(a) 48.2 (b) 51.9		Videostroboscopy, VHI, MPT, jitter, shimmer, HNR	>3 y Mean (a/b): 50.6/45.5 m
Wang et al., 2020, China [38]	1994–2017	RC	(a) 37, (b) 16 ^11^	(a+b) ARA:53	(a) 44.8 (b) 47.5	(a) 28, (b) 12	Videostroboscopy, LEMG, GRB, HNR, jitter, shimmer, MPT	1y
Yuan et al., 2020, China [39]	2004–2018	R	37	SVR:17, ARA:8, FNG:4	48	16	Laryngoscopy, GRBAS, MPT	1–15 y, mean: 8.5 y
Wu et al., 2020, China [40]	2009–2020	R	13	DA:13	39	11	GRBAS	6 m
Wan Mansor et al., 2021, Malaysia [41]		R	3	ARA:2, DA:1	40	2	Laryngoscopy, MPT, VHI-10, shimmer, jitter, HNR, LEMG	12 m

^1^ (a) primary RLN neurorrhaphy group, (b) patients with UVFP, and (c) general population. ^2^ (a) primary neurorrhaphy and (b) RLN injury with no intervention. ^3^ (a) primary neurorrhaphy, (b) RLN injury with no intervention, and (c) arytenoid abduction. ^4^ (a) primary neurorrhaphy, (b) general population, and (c) patients with UVFP. ^5^ (a) primary neurorrhaphy with presence of pre-op UVFP and (b) primary neurorrhaphy without pre-op UVFP. ^6^ (a) primary neurorrhaphy and (b) non-neurorrhaphy group. ^7^ (a) primary neurorrhaphy, (b) general population, and (c) RLN injury with no intervention. ^8^ (a) primary neurorrhaphy with presence of pre-op UVFP, (b) primary neurorrhaphy without pre-op UVFP, and (c) type 1 thyroplasty. ^9^ (a) primary neurorrhaphy and (b) RLN injury with no intervention. ^10^ (a) primary neurorrhaphy and (b) injection laryngoplasty. ^11^ (a) primary neurorrhaphy with presence of pre-op UVFP and (b) primary neurorrhaphy without pre-op UVFP. ARA: Ansa to Recurrent Nerve Anastomosis, DA: Direct Anastomosis, FNG: Free Nerve Graft. GRBAS: Grade Roughness Breathiness Authenticity Strain Scale, GRB: Grade Roughness Breathiness (Hirano Scale), HNR: Harmonics to Noise Ratio, IL: Injection Laryngoplasty, LEMG: Laryngeal Electromyography, m: Months, MFR: Maximum Flow Rate, MPT: Maximum Phonation Time, P: Prospective, PC: Prospective Comparative, PEI: Phonation Efficacy Index, R: Retrospective, RC: Retrospective Comparative, SVR: Selective Vagus to RLN Anastomosis, UVFP: Unilateral Vocal Fold Palsy, VHI: Voice Handicap Index, VRA: Vagus to Recurrent Nerve Anastomosis, and y: years.

**Table 4:** Overview of the studies’ outcomes.

Studies	Outcome
Miyauchi et al., 1998 [25]	Improvement in MPT at 2–5 m post-op. MPT stable at 12 m, comparable to general population, better than UVFP group (mean 26.2 ± 13 in men *p* < 0.02 and 17.2 ± 5.6 secs on women *p* < 0.0001 ss). Laryngoscopy: fixation of VC in middle position except for a few cases of minimal mobility, no atrophy, normal tone, minimal glottal gap during phonation.
Chou et al., 2003 [26]	Improvement of all parameters studied at 6 m post-op. Improvement in the GRBAS scale and aspiration assessment at 6 m compared to 3 m post-operatively. In 11 out of 12 who underwent neurorrhaphy reduction of glottal gap, immobility of the VC without atrophy of it (nss).
Yumoto et al., 2006 [27]	Minimization or elimination of the glottal gap during phonation. Improvement of all acoustic analysis parameters.
Miyauchi et al., 2009 [18]	1 y post-op MPT and PEI were found greater than UVFP group (statistical significance: MPT 1 year post-op 20.9 ± 11.7 sec in men and 18.8 ± 6.6 in women *p* < 0.05 and PEI 7.22 ± 2.9 *p* < 0.0001) and comparable to healthy subjects.
Sanuki et al., 2010 [28]	Improvement of GRBAS post-operatively, reduction or elimination of glottal gap during phonation, restoration of mucosal wave post-op. Improvement of MPT and MFR.
Rohde et al., 2012 [29]	Improvement in GRBAS and VHI. Videostroboscopy: middle position of the VC (good tone, normal volume) with adequate glottal closure during phonation.
Hong et al., 2014 [30]	Improvement of all parameters at 12 m post-operatively. Improvement at 12 m when compared to results at 3 m post-operatively (statistical significance: 4.4 ± 0.84 sec vs. 11.7 ± 0.95 *p* = 0.002).
Lee et al., 2014, [31]	Improvement in VHI at 6 m and remaining at these levels at 12 m post-op, (statistical significance: VHI 6 m post-op 38.2 ± 24.1 vs. 84.89 ± 17, *p* < 0.05) improvement in glottal closure and mucosal wave. Improvement in shimmer, HNR, and MPT at 12 m post-op (statistical significance: HNR 6 m post-op 34.9 ± 1.2 vs. 16.8 ± 5.3, MPT at 6 m post-op 10.40 ± 2.59 vs. 6.69 ± 3.01, and shimmer 5.11 ± 4.09 vs. 8.05 ± 5.35 *p* < 0.05) remaining stable at 24 m.
Kumai et al., 2015 [32]	Improvement of videostroboscopic findings: wave width, glottal closure, and periodicity at 12 m post-op. Improvement in MPT, MFR, jitter, and shimmer 12 m post-op (statistical significance: MPT 22 vs. 8.9 *p* < 0.05, MFR 128 vs. 247 *p* < 0.05, jitter 1.1 vs. 3.5 *p* < 0.05, and shimmer 5 vs. 9.1 *p* < 0.05).
Dzodic et al., 2015 [33]	Preservation of VC’s tone post-op and achievement of near-normal phonation 12 m post-op. Improvement in vocal assessment scale and vocal fold immobility.
Yoshioka et al., 2016 [34]	Normal mobility of the VC was not achieved. Increase in MPT 12 m post-op (statistical significance: mean MPT 15 *p* < 0.0005). MFR improvement in 12 m post-op.
Iwaki et al., 2016 [35]	Fixation of the VC in median or paramedian position but no atrophy occurred (normal tone). Normal glottal closure was achieved. Post-op improvement in PEI, MPT, HNR, jitter%, and shimmer% after 12 m.
Gurrado et al., 2018 [36]	Improved GRBAS and subjective assessment for aspiration. Fixation of the VC in a middle position without atrophy with good tone.
Lee et al., 2018 [37]	Improvement in VHI and laryngoscopic reduction in glottal gap, improvement in mucosal wave at 12, 24, and 36 m post-op (statistical significance: VHI 36 m post-op 41.8 ± 25.2 vs. 24 m post-op 37.1 ± 21.5 vs. 12 m post-op 34.6 ± 19.9; glottis gap 2.2 ± 0.7 vs. 2.4 ± 0.5 vs. 2.6 ± 0.6; mucosal wave 2.1 ± 0.7 vs. 2.4 ± 0.5 vs. 2.5 ± 0.7; MPT 10.79 ± 1.64 vs. 10.53 ± 1.18 vs. 10.58 ± 2.29; jitter 0.95 ± 0.72 vs. 1.30 ± 0.52 vs. 1.52 ± 0.92; shimmer 2.87 ± 0.96 vs. 2.92 ± 0.96 vs. 2.86 ± 0.99; HNR 21.8 ± 3.7 vs. 22.2 ± 4.2 vs. 26.1 ± 3.7 all with *p* < 0.05). Improvement in MPT, jitter%, shimmer%, and HNR at 12, 24, and 36 m post-op (statistical significance).
Wang et al., 2020 [38]	Improvement of the GRB scale at 12 m post-op (nss). Videostroboscopy: elimination of glottal gap, symmetry, and regularity in the VC vibration but immobile, fixation in middle or paramedian position. Improvement in jitter, shimmer, HNR, and MPT at 1 y post-op. Improvement in VMUR recorded by LEMG at 12 m post-op (deterioration had occurred at the 1st month).
Yuan et al., 2020 [39]	Improvement in GRBAS and laryngoscopy. VC immobility with normal tone, no atrophy, and minimal glottal gap. Improvement in MPT from 1st to 3rd months of post-op.
Wu et al., 2020 [40]	Improvement on GRBAS scale in all patients.
Wan Mansor et al., 2021 [41]	Improvement in VHI-10 at 3 m post-op. Laryngoscopy: immobility of the VC with fixation in the middle position. Improvement and return to normal levels in jitter%, shimmer%, HNR, and MHF in all post-op measurements except for the 3rd m.

FNG: Free Nerve Graft; GRBAS: Grade–Roughness–Breathiness–Authenticity–Strain Scale; LEMG: Laryngeal Electromyography; m: months; MFR: Mean Flow Rate; MPT: Maximum Phonation Time; nss: no statistical significance; PEI: Phonation Efficacy Index; ss: statistical significance; UVFP: Unilateral Vocal Fold Palsy; VC: Vocal Cord; VHI: Voice Handicap Index; VMUR: Voluntary Motor Unit Recruitment; and y: year.

**Table 5:** Assessments of studies included using the Newcastle–Ottawa Scale.

	Selection				Comparability	Outcome			Total Score
	I1	I2	I3	I4	I5	I6	I7	I8	
Miyauchi et al., 1998 [25]	1	0	1	1	1	1	1	1	7
Chou et al., 2003 [26]	1	0	1	1	1	1	0	0	5
Yumoto et al., 2006 [27]	1	0	1	1	1	1	1	1	7
Miyauchi et al., 2009 [18]	1	1	1	1	1	1	1	1	8
Sanuki et al., 2010 [28]	1	0	1	1	1	1	1	0	6
Rohde et al., 2012 [29]	1	0	1	1	1	1	1	1	7
Hong et al., 2014 [30]	1	1	1	1	1	1	1	1	8
Lee et al., 2014 [31]	1	0	1	1	1	1	1	1	7
Kumai et al., 2015 [32]	1	0	1	1	1	1	1	1	7
Dzodic et al., 2015 [33]	1	0	1	1	1	0	1	1	6
Yoshioka et al., 2016 [34]	1	1	1	1	1	1	1	1	8
Iwaki et al., 2016 [35]	1	1	1	1	1	1	0	1	7
Gurrado et al., 2018 [36]	1	1	1	1	1	0	1	0	6
Lee et al., 2018 [37]	1	1	1	1	1	1	1	1	8
Wang et al., 2020 [38]	1	1	1	1	1	1	1	1	8
Yuan et al., 2020 [39]	1	0	1	1	1	1	1	1	7
Wu et al., 2020 [40]	1	0	1	1	1	0	1	0	5
Wan Mansor et al., 2021 [41]	1	0	1	1	1	1	1	1	7

Items 1–8: I1: Representativeness of the exposed cohort; I2: Selection of the non-exposed cohort; I3: Ascertainment of exposure; I4: Demonstration that outcome of interest was not present at the start of the study; I5: Comparability of cohorts based on the design or analysis; I6: Assessment of outcome; I7: Was follow-up long enough for outcomes to occur; I8: Adequacy of follow-up of cohorts.

## Data Availability

No new data were created or analyzed in this study. Data sharing is not applicable to this article.

## Supplementary Materials

The following supporting information can be downloaded at: https://www.mdpi.com/article/10.3390/jcm12031212/s1, PRISMA 2020 checklist.

## Abbreviations

ARA	Ansa to recurrent nerve anastomosis
DA	Direct Anastomosis
FNG	Free Nerve Graft
GRB	Grade–Roughness–Breathiness (Hirano Scale)
GRBAS	Grade–Roughness–Breathiness–Authenticity–Strain Scale
HNR	Harmonics to Noise Ratio
IL	Injection Laryngoplasty
LEMG	Laryngeal Electromyography
m	months
MFR	Maximum Flow Rate
MPT	Maximum Phonation Time
nss	no statistical significance
P	Prospective
PC	Prospective Comparative
PEI	Phonation Efficacy Index
R	Retrospective
RC	Retrospective Comparative
RLN	Recurrent Laryngeal Nerve
ss	statistical significance
SVR	Selective Vagus to RLN Anastomosis
UVFP	Unilateral Vocal Fold Palsy
VC	Vocal Cord
VHI	Voice Handicap Index
VMUR	Voluntary Motor Unit Recruitment
VRA	Vagus to Recurrent Nerve Anastomosis
y	years
